# Protective effects of berberine on doxorubicin-induced nephrotoxicity in mice

**DOI:** 10.1186/1479-5876-10-S2-A66

**Published:** 2012-10-17

**Authors:** Xiaoyan Zhao, Nannan Tong

**Affiliations:** 1 College of Pharmaceutical Sciences, Southwest University, Chongqing 400716, China

## Background and purpose

Doxorubicin, a very potent and often used anti-cancer drug, is largely limited due to the dose-related toxic effects. The present study investigated whether berberine, a natural product alkaloid, can reduce the renal injury induced by doxorubicin.

## Experimental approach

Mice of either gender were randomly divided into four groups: the control group, doxorubicin group, berberine group, and berberine+doxorubicin group. In the tests, body weight, general condition and mortality of the mice were observed, and serum blood urea nitrogen (BUN) and serum albumin (Alb) levels were determined to evaluate renal function. Furthermore, the renal was excised for determination of the weight changes, as well as histopathological analysis in the tissues.

## Key results

Mortality rate and significant decline in body weight, and increased blood urea nitrogen (BUN) and serum albumin (Alb) levels were observed in doxorubicin-treated mice. These changes were significantly prevented by pretreatment with berberine. Histopathological studies showed that doxorubicin caused structural injuries, such as glomerular, tubular epithelial alterations and interstitial edema in the renal. These histopathological changes were largely attenuated by berberine pretreatment.

## Conclusions

These findings indicate that berberine could play an important role in ameliorating the doxorubicin-induced toxicity.

